# New data on species diversity of Annelida (Oligochaeta, Hirudinea) in the Kharbey lakes system, Bolshezemelskaya tundra (Russia)

**DOI:** 10.3897/zookeys.910.48486

**Published:** 2020-02-10

**Authors:** Maria A. Baturina, Irina A. Kaygorodova, Olga A. Loskutova

**Affiliations:** 1 Institute of Biology of Komi Scientific Centre of the Ural Branch of the Russian Academy of Sciences, 28 Kommunisticheskaya Street, 167982 Syktyvkar, Russia Komi Scientific Centre, Ural Branch of the Russian Academy of Sciences Syktyvkar Russia; 2 Limnological Institute, Siberian Branch of Russian Academy of Sciences, 3 Ulan-Batorskaya Street, 664033 Irkutsk, Russia Limnological Institute, Siberian Branch of Russian Academy of Sciences Irkutsk Russia

**Keywords:** Annelid biodiversity, lakes, leeches, northwest Russia, oligochaetes, tundra

## Abstract

One of the features of the tundra zone is the diversity of freshwater bodies, where, among benthic invertebrates, representatives of Annelida are the most significant component in terms of ecological and species diversity. The oligochaete and leech faunas have previously been studied in two of the three largest lake ecosystems of the Bolshezemelskaya tundra (the Vashutkiny Lakes system, Lake Ambarty and some other lakes in the Korotaikha River basin). This article provides current data on annelid fauna from the third lake ecosystem in the region, Kharbey Lakes and adjacent water bodies. The annelid fauna includes 68 species, including 51 oligochaete species, and 17 species of leeches. For each species, we give information on currently recognised classification, taxonomic synonymy, geographical distribution, findings of the species within the Russian tundra, and brief ecological characteristics.

## Introduction

The tundra covers an area of approximately 15 % of the entire territory of Russia, along the entire coast of the Arctic Ocean, from the Finland border in the west to the Bering Strait in the east. Bolshezemelskaya tundra is a vast plain with an area of 1,660 km^2^ located between the Pechora and Usa rivers (in the west and south) and the Ural Mountains in the east, adjacent to the Barents Sea in the north. Hills with prevailing heights of 100–150 m and moraine ridges of up to 250 m high characterises its relief. The main part of the Bolshezemelskaya tundra is occupied by permafrost. Here, peat bog and silt-marsh soil types prevail; in the south, there are weakly podzol-gley soils. The climate is subarctic, with long cold winters and short cool summers. Many rivers which are tributaries of the Pechora and Usa flow through the plain. The main watershed is located in its central part, with the largest lakes systems in the east of the Bolshezemelskaya tundra: the Vashutkiny, Padimeyskiye, Kharbey Lakes and the other lakes of the Korotaikha River basin.

**Figure 1.**
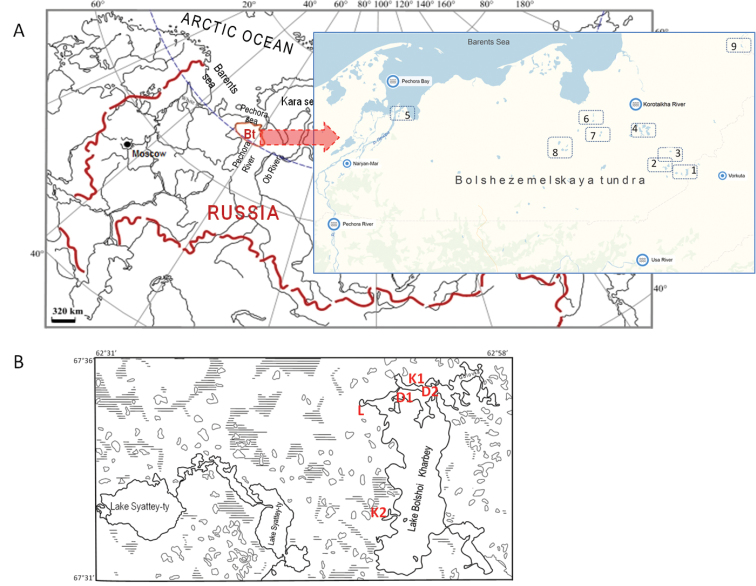
Geographical location of the study region. (**A**) The map of Russia showing location of the Bolshezemelskaya tundra (Bt). The numbers indicate the major studied systems of tundra lakes: 1 – the Kharbey lake system, 2 – the Padimeyskiye lake system, 3 – Lake Ambarty, 4 – the Vashutkiny lake system; 5 – Lakes of the Pechora River Delta; 6 – lakes of the More-yu River basin (lower reaches); 7 – lakes of the More-yu River basin (upper reaches); 8 – lakes of central part of Bt; 9 – Lake Ngosovey. (**B**) Insert showing the Kharbey Lake system: K1, K2, D1, D2, and L are small lakes adjacent to Lake Bolshoy Kharbey.

In this study, we focused on the waters of the Kharbey system, the main element of which is Bolshoy Kharbey Lake, located in the headwaters of the River Kharbeytyvis, the right tributary of the Seyda River. In addition, this system includes the lakes Golovka and Maliy Kharbey. The larger lakes are interconnected by natural channels and are surrounded by numerous shallow adjacent lakes and have a glacial origin. The Bolshoy Kharbey is the largest lake of the system (Vinberg and Vlasova 1976); its area is 21 km^2^, and depth is up to 18 m (70% of the lake has a depth of 1-6 m). The shoreline of the lake is indented, forming bays and gulfs. The lakeshore habitats are dry, mostly low, and peaty in some places. Bottom sediments in littoral habitats are pebble-boulder or sandy, in deeper water, the sandy substrate is covered with silt. There are many temporary water bodies in the catchment area of the Kharbey lakes. To the west of the Kharbey lakes, there is Lake Syattey-ty, which consists of two connected reservoirs (Fig. 1). The area of the larger lake, Bolshoy Syattey-ty, is 7.4 km^2^; the catchment area is 66.2 km^2^. Gradually-sloping shores located near numerous small lakes are overgrown with sedge and willow; depth of these smaller lakes is 3.2-7.4 m; bottom sediments are mostly sandy and sometimes silty.

The first studies of the Kharbey system were carried out in 1965–1972 in order to evaluate the productivity and environmental features of lakes (Vinberg and Vlasova 1976). In these lakes, as in other lake systems of the Bolshezemelskaya tundra, a diverse and unique flora and fauna was described. However, there are no data on the species composition of annelids. Later, Zaloznyj (1978) found six species of leeches in this lake. In the late 1990s and early 2000s, complex studies of the ecosystem state, including the structural characteristics of benthic and plankton communities, were conducted in the Kharbey lakes, and these studies provided data on the faunal composition of various taxonomic groups, including Annelida (Fefilova et al. 2014, Baturina et al. 2014a).

The aim of this study is to further investigate the annelid species diversity and spatial distribution in the Kharbey Lakes system, as one of the largest systems of the Bolshezemelskaya tundra, combining the available literature data with new information about the Annelida fauna.

## Materials and methods

Previously published information and an extensive collection of new specimens from fresh water bodies of the Kharbey Lake area collected by M. Baturina and O. Loskutova in 1998-99, 2009, 2010, and 2012 were used in this study. Within this study, the following water bodies of the Kharbey lakes system (Bolshezemelskaya tundra) were investigated: Lake Bolshoy Kharbey, Lake Golovka, and some unnamed smaller lakes adjacent to Lake Bolshoy Kharbey, arbitrarily identified as K1, K2, L, D1, and D2 (Fig. 1B). Additionally, 41 small temporary habitats (including swamps, depressions, and ponds), located within the catchment area of B. Kharbey, were investigated. In 2014, hydrobiological material was collected in Lake Syattey-ty (Bolshezemelskaya tundra) and small water bodies in its watershed. Main sampling locations are shown in Fig. 1.

Oligochaete samples were taken with a Petersen grab (sampling area 400 cm^2^) on soft substrates and with a handle blade trawl (Zinchenko et al. 2014) on gravel substrates. Since the common hydrobiological equipment (sweep net, dredge, scraper, bottom grab, etc.) is often ineffective in collecting parasitic and predatory leeches, we inspected various aquatic plants and animals, as well as submerged objects (rotten wood, driftwood, snags, stones, etc.) for attached hirudinids. Some leeches were picked out from zoobenthic samples. In most cases, piscivorous leeches were collected directly from captured living hosts.

Newly collected specimens were fixed and kept in 80% ethanol solution. Morphological analysis was performed using a stereomicroscope MSP-2 var. 2 (LOMO) and compound microscope Leica DM 4000. The worm species determinations were based on existing taxonomic keys (Chekanovskaya 1962; Lukin 1976; Nesemann and Neubert 1999; Timm 2009) in accordance with the present-day classification of each group. As to names of higher oligochaete taxa, there is still no unanimous opinion; therefore, we left them as in Timm (2009). Voucher specimens were deposited at the Institute of Biology, Syktyvkar (Oligochaeta) and Limnological Institute, Irkutsk (Acanthobdellida and Hirudinea).

### Data resources

The data underpinning the analysis reported in this paper are deposited at GBIF, the Global Biodiversity Information Facility, and are available at https://doi.org/10.15468/b24asb.

## Results

This research describes the Annelida fauna of one of the largest lakes systems of Bolshezemelskaya tundra, Kharbey lakes. We show a list of oligochaete species (Oligochaeta), leeches (Hirudinea) and leech-like parasites (Acanthobdellida) for various types of water bodies of the Kharbey lakes system and the nearby Syattey-ty lakes system, and revise taxonomic and nomenclatural changes since the last fauna surveys in the lakes of Bolshezemelskaya tundra (Finogenova 1962, 1966; Lukin 1962, 1966; Zaloznyj 1978; Popchenko 1978, 1988).

The Oligochaeta fauna of tundra water bodies is considered to be significantly poorer in comparison with the nearby northern areas, such as the Kola Peninsula (Jakovlev 1982; Popchenko 1988). The main components of the fauna were cosmopolitan species or species that are widespread in the Palaearctic or Holarctic zoogeographic regions. Fifty-one oligochaete species were found in Lake Bolshoy Kharbey, its accessory water bodies and temporary watersheds; 15 of these were not previously observed in the systems of large lakes of the Bolshezemelskaya tundra, such as Vashutkiny and Ambarty. However, the overlap of the oligochaete species composition in all three lakes systems was more than 50%. Despite this taxonomic diversity, the list of known widespread species is relatively short in most water bodies of the Kharbey Lakes system: there were only three species (*Lumbriculus variegatus* (Müller), *Tubifex tubifex* (Müller) and *Spirosperma ferox* Eisen) recorded in the bulk of the studied water bodies, and 33 species were only observed once.

Oligochaete worms dominated the total numbers and biomass of zoobenthos at different depths on all the studied sediment types in the Kharbey system. The average abundance of oligochaetes showed the highest values in the upper and lower littoral zones (0–3–6 m), whereas on silts in the profundal zone (depths 6–9 m, max 18 m), the average abundance of the group was half as great. In most biotopes, *S. ferox* and *T. tubifex* were among the dominant species. On the gravel-pebble substrates of the littoral zone, subdominants included *Nais barbata* Müller, *Uncinais uncinata* (Øersted), Tubificinae gen. sp. juv., and Enchytraeidae gen. sp. juv.; the same species were also dominant on sandy sediment, along with *Cognettia glandulosa* (Michaelsen), *L. variegatus* and *Piguetiella blanci* (Piguet). On clayey substrate of the littoral and sublittoral zones, there was a group of minor species: *Chaetogaster diaphanus* (Gruithuisen), *Nais alpina* Sperber, *Nais bretscheri* Michaelsen, *Nais pseudobtusa* Piguet, *U. uncinata*, *S. ferox* and *Lophochaeta ignota* (Štolc), Enchytraeidae gen. sp. juv.; on profundal silts were *N. pseudobtusa* and *Vejdovskyella comata* (Vejdovský).

The species distribution of oligochaetes in lakes is usually determined by the substrate (O’Toole et al. 2008) and the oxygen regime (Timm 1987). At the same time, high diversity of naidids is probably associated with the variety of sediments and aeration in the littoral zone; as opposed to the profundal zone, where the dominants are tubificines. The complex of species *S. ferox* – *T. tubifex*, typical for the profundal zone of the most lakes, varies among the lakes under study: *S. ferox* remains the dominant species in the littoral and sublittoral zones, and *T. tubifex* descends to the less aerated deep-water zone, consistent with previous observations for small oligotrophic profundal lakes (Timm 1987).

The leech and leech-like taxonomic diversity includes 17 species belonging to three orders (Acanthobdellida Grube, Rhynchobdellida Blanchard and Arhynchobdellida Blanchard), five families (Arhynchobdellidae Grube, Glossiphoniidae Vaillant, Piscicolidae Johnston, Erpobdellidae Blanchard and Haemopidae (Richardson)), and nine genera (*Acanthobdella* Grube, *Glossiphonia* Johnston, *Helobdella* Blanchard, *Hemiclepsis* Vejdovský, *Theromyzon* Philippi, *Piscicola* de Blainville, *Cystobranchus* Diesing, *Erpobdella* de Blainville, and *Haemopis* (Savigny)). We collected only three of the five leech species recorded by Zaloznyj (1978) in the Kharbey lakes: *Glossiphonia complanata* (Linnaeus), *Glossiphonia concolor* (Apathy), and *Piscicola geometra* (Linnaeus). Of the newly recorded species, the tundra piscine parasite *P. geometra* has a specific segmentally repeated geometrical pattern of greenish-brown pigment on the dorsal side, but smaller body size dimensions in comparison with typical representatives of the species. Moreover, the new checklist includes 5 species (*Acanthobdella peledina* Grube, *Theromyzon tessulatum* (Müller), *Piscicola* sp., *Erpobdella monostriata* (Lindenfeld et Pietruszynski), and *Erpobdella* sp.) recorded for the first time for the Kharbey system. Among these, there are two potentially new species (*Erpobdella* sp. and *Piscicola* sp.), which differ from published descriptions. At the same time, eight species noted by previous authors (Lukin 1976; Zaloznyj 1978) from the Kharbey lakes – *Glossiphonia verrucata* (Müller), *Hemiclepsis marginata* (Müller), *Helobdella stagnalis* (Linnaeus), *Theromyzon maculosum* (Rathke), *Erpobdella octoculata* (Linnaeus), *Erpobdella nigricollis* (Brandes), *Erpobdella testacea* (Savigny), and *Haemopis sanguisuga* (Linnaeus) – were not found in our samples, although they may, supposedly, live there as noted by previous authors (Lukin 1976; Zaloznyj 1978). The burbot leech *Cystobranchus mammillatus* Malm, which we found in the Pechora River, is quite likely to be present in the Kharbey system. Despite earlier records listing two macrophagous leeches, *E. octoculata* and *E. nigricollis*, as the most numerous species (Lukin 1976), our samples did not contain these species. Although we did not find *E. testacea*, similar leeches, corresponding to *E. monostriata* in recent taxonomic revisions (Agapow and Bielecki 1992; Nesemann and Neubert 1999; Utevsky et al. 2015), were numerous numerous in Lake Bolshoy Kharbey and small lakes. Despite having a wide distribution range, *G. verrucata*, which is quite sensitive to habitat quality, has seemingly become too scarce in Western Europe; and we did not find this species in the northwestern part of Russia. The absence of the two most common Palaearctic species, *H. stagnalis* and *H. marginata*, in our samples from Kharbey lakes is very strange and unexpected. These findings can probably be attributed to sampling methods that were not focused on leeches. The “large false horse” leech *H. sanguisuga* is especially difficult to find since it often leaves water and lays its cocoons in moist soil near the shore (up to a vertical 2-3 cm above the water surface) (Nesemann and Neubert 1999). The presence of the waterfowl parasite *T. maculosum* in the Kharbey area was highly expected due to its previous findings in different lakes of the Komi region (Lukin 1957, 1962), although this discrepancy could be due to prior misidentifications.

Information on exact systematic position, geographical distribution and brief ecological characteristics for each Annelida species is given in the list below.

### Systematics

#### Phylum Annelida Lamarck, 1809

##### Class Clitellata Michaelsen, 1919

###### Subclass Oligochaeta Grube, 1850

####### Order Tubificida Brinkhurst, 1982

######## Family Naididae Ehrenberg, 1828

######### Subfamily Naidinae Ehrenberg, 1828

########## Genus: *Amphichaeta* Tauber, 1879

########### 1. Amphichaeta leydigi

Taxon classificationAnimaliaTubificidaNaididae

Tauber, 1879

5BC01B7A-9E28-534C-9C19-F5F2D0069916

############ Geographic distribution.

Holarctic species. In the Russian tundra: Murmansk Region, coast of the White Sea (Timm and Abarenkov 2018).

############ Location.

Lake Bolshoy Kharbey (67°32'48.3"N, 62°53'49.7"E; 67°34'34.3"N; 62°52'17.4"E).

############ Ecology.

The species was recorded on clay, silted sand, large pebbles, often in moss and algal cover (depth 0.8 m, maximum up to 5.2 m).

########## Genus *Arcteonais* Piguet, 1928

########### 2. Arcteonais lomondi

Taxon classificationAnimaliaTubificidaNaididae

(Martin, 1907).

AAB302A5-6568-52E7-A9D8-C53CEAF938CF

Stylaria lomondi Martin, 1907
Stylaria brevirostris Wolf, 1928

############ Geographic distribution.

Holarctic species. In the Russian tundra: Murmansk Region (Timm and Popchenko 1978), the Pechora River delta (Baturina 2018), the Vashutkiny lakes system (Finogenova 1966).

############ Location.

Lake Bolshoy Kharbey (67°34'34.3"N, 62°52'17.4"E); Lake Golovka (67°36'0.6"N, 62°55'28.6"E).

############ Ecology.

The species was recorded on silt or silted sand (depth 6.2–7.5 m).

########## Genus *Bratislavia* Košel, 1976

########### 3. Bratislavia palmeni

Taxon classificationAnimaliaTubificidaNaididae

(Munsterhjelm, 1905).

394A96BD-12E5-5563-9739-79160852008A

Naidium palmeni Munsterhjelm, 1905
Pristina elegans Finogenova, 1966
Pristina napocensis Pop, 1973

############ Geographic distribution.

Europe. In the Russian tundra: Lake Balban-ty (Finogenova 1966).

############ Location.

Lake Bolshoy Kharbey (67°31'49.9"N, 62°52'40.1"E), temporary pond near Kharbey (67°58'N, 62°34'60"E).

############ Ecology.

The species lives in lake on clayey sediment (at a depth of up to 5.8 m), and temporary ponds within wetlands.

########## Genus *Chaetogaster* Baer, 1827

########### 4. Chaetogaster diaphanus

Taxon classificationAnimaliaTubificidaNaididae

(Gruithuisen, 1828)

5D4E1371-09E2-55BB-8768-8F42E6E270DD

Nais diaphanus Gruithuisen, 1828

############ Geographic distribution.

Cosmopolitan species. In the Russian tundra: Murmansk Region (Finogenova 1975; Timm and Popchenko 1978), the Solovetsky Islands (Popchenko 1972), Vaygach Island (Leshko et al. 2008), the Pechora River delta (Baturina 2018), the Vashutkiny lakes system (Finogenova 1966), Lake Ambarty and some other lakes in the Korotaikha River basin (Popchenko 1978), lakes in the Kara River basin, lakes in the More-yu River basin, Lake Bolshoy Ngosovey (Baturina and Loskutova 2010), the Ob River delta (Timm and Abarenkov 2018), the northern part of Western Siberia (Zaloznyj 1984), the Yamal Peninsula (Stepanov 2016).

############ Location.

Lake Bolshoy Kharbey (67°34'34.3"N, 62°52'17.4"E; 67°33'48.2"N, 62°55'2.6"E; 67°32'49.4"N, 62°53'6.6"E; 67°31'49.9"N, 62°52'40.1"E); Lake L (67°35'46"N, 62°49'44.8"E); Lake K1 (67°36'17.6"N, 62°52'35"E); Lake Golovka (67°35'50"N, 62°55'25.3"E).

############ Ecology.

The species inhabits various sites with rocky, sandy, and vegetative substrates (depths 0.3–4.2 m).

########### 5. Chaetogaster diastrophus

Taxon classificationAnimaliaTubificidaNaididae

(Gruithuisen, 1828)

91CD2926-7E02-5C43-B577-DDAC17756C61

Nais diastrophus Gruithuisen, 1828
Chaetogaster palustris Pointner, 1914

############ Geographic distribution.

Cosmopolitan species. In the Russian tundra: Murmansk Region (Finogenova 1975; Timm and Popchenko 1978), the Vashutkiny lakes system (Finogenova 1966), Lake Ambarty and some other lakes in the Korotaikha River basin (Popchenko 1978), lakes in the Kara River basin, lakes in the Malaya Usa River basin (Baturina and Loskutova 2010).

############ Location.

Lake Bolshoy Kharbey (67°34'3.5"N, 62°52'17.9"E; 67°32'48.3"N, 62°53'49.7"E; 67°33'48.2"N, 62°55'2.6"E; 67°34'34.3"N, 62°52'17.4"E).

############ Ecology.

The species was recorded on stones with algal cover and on sand, at depths of 0.5–3.8 m.

########### 6. Chaetogaster setosus

Taxon classificationAnimaliaTubificidaNaididae

Svetlov, 1925

4F93BB05-16B9-58AA-8B9F-39D44EF8DAF1

############ Geographic distribution.

Holarctic species. In the Russian tundra: Murmansk Region (Timm and Popchenko 1978), and the Pechora River delta (Baturina 2018).

############ Location.

Lake Bolshoy Kharbey (67°32'44.2"N, 62°55'22.3"E).

############ Ecology.

The species of rare in studied region; it was found on silted sand at a depth of 3.8 m.

########## Genus *Nais* Müller, 1774

########### 7. Nais alpina

Taxon classificationAnimaliaTubificidaNaididae

Sperber, 1948

13CFEDC6-3488-5101-AFBC-B70F549A5E6C

############ Geographic distribution.

In Europe and North America (Great Lakes). In the Russian tundra: Murmansk Region (Timm and Popchenko 1978), Vaygach Island (Leshko et al. 2008), lakes in the More-yu River basin, lakes in the Kara River basin (Baturina and Loskutova 2010), lakes in the Malaya Usa River basin (Baturina et al. 2014b).

############ Location.

Lake Bolshoy Kharbey (67°34'3.5"N, 62°52'17.9"E; 67°34'34.3"N, 62°52'17.4"E; 67°31'49.9"N, 62°52'40.1"E; 67°32'49.4"N, 62°53'6.6"E; 67°31'49.9"N, 62°52'40.1"E); Lake Golovka (67°35'50"N, 62°55'25.3"E; 67°36'9.4"N, 62°56'39.9"E).

############ Ecology.

The species inhabits stones with algal cover or sand with detritus (depth 0.2–1.3 m).

########### 8. Nais barbata

Taxon classificationAnimaliaTubificidaNaididae

Müller, 1774

1A906AA7-3AEB-5F96-AE1A-3D99C39D335A

############ Geographic distribution.

Holarctic species. Sino-Indian Region and Australia. In the Russian tundra: Murmansk Region (Stalmakova 1974), the Pechora River delta (Baturina 2018), Vaygach Island (Leshko et al. 2008), the Vashutkiny lakes system (Finogenova 1966), Lake Ambarty and some other lakes in the Korotaikha River basin (Popchenko 1978), Lake Bolshoy Ngosovey and lakes in the More-yu River basin (Baturina and Loskutova 2010), the North of Western Siberia (Zaloznyj 1984), the Anadyr River basin (Morev 1983b), the Yamal Peninsula (Stepanov 2017), the Kolyma River basin (Morev 1983a, Morev et al. 1985).

############ Location.

Lake Bolshoy Kharbey (67°32'48.3"N, 62°53'49.7"E; 67°32'49.4"N, 62°53'6.6"E; 67°33'48.2"N, 62°55'2.6"E; 67°34'34.3"N, 62°52'17.4"E; 67°35'27.5"N, 62°55'30.7"E); Lake Golovka (67°36'9.4"N, 62°56'39.9"E).

############ Ecology.

The species was recorded on sands, stones with algal cover, as well as on clay and submerged macrophytes (depth 0.5–2.8 m).

########### 9. Nais behningi

Taxon classificationAnimaliaTubificidaNaididae

Michaelsen, 1923

B5F5152A-D14F-534A-AB56-2ACFA964425B

############ Geographic distribution.

Holarctic species. In the Russian tundra Murmansk Region (Veselov 1977; Timm and Popchenko 1978), the Pechora River delta (Baturina 2018), lakes in the More-yu River basin (Baturina and Loskutova 2010).

############ Location.

Lake Bolshoy Kharbey (67°31'21.1"N, 62°53'28.6"E; 67°32'22.1"N, 62°52'10.7"E).

############ Ecology.

Within the studied water bodies, *N. behningi* was found on stony sediments with moss cover (depth 0.7–2.0 m).

########### 10. Nais bretscheri

Taxon classificationAnimaliaTubificidaNaididae

Michaelsen, 1899

D3CC5DC6-9953-5213-8D97-891DD9D0A670

############ Geographic distribution.

Holarctic.

############ Location.

Lake Bolshoy Kharbey (67°32'49.4"N, 62°53'6.6"E; 67°31'38"N, 62°53'2.8"E; 67°32'49.9"N, 62°53'40.1"E).

############ Ecology.

The species inhabits stony ground and mosses among large pebbles, typically at a depth of up to 1.0 m; it occasionally occurred at a depth of 2.5 m.

########### 11. Nais communis

Taxon classificationAnimaliaTubificidaNaididae

Piguet, 1906

731A3A90-9C3A-59CD-A55C-520337417D42

############ Geographic distribution.

Cosmopolitan species. In the Russian tundra: Murmansk Region (Stalmakova 1974; Finogenova 1975; Timm and Popchenko 1978), the Pechora River delta (Baturina 2018), Vaygach Island (Leshko et al. 2008), the Vashutkiny lakes system (Finogenova 1966), lakes in the More-yu River basin, Lake Bolshoy Ngosovey (Baturina and Loskutova 2010), Lake Ambarty and some other lakes in the Korotaikha River basin (Popchenko 1978), lakes in the Kara River basin, lakes in the Malaya Usa River basin, lakes in the Bolshaya Usa River basin (Baturina et al. 2014b), the Yamal Peninsula (Stepanov 2016), northern part of Western Siberia (Zaloznyj 1984), the Kolyma River basin (Morev et al. 1985).

############ Location.

Lake Bolshoy Kharbey (67°32'44.2"N, 62°55'22.3"E; 67°32'49.9"N, 62°53'40.1"E; 67°34'3.5"N, 62°52'17.9"E); Lake K1 (67°36'27.6"N, 62°51'58.4"E); Lake D1 (67°35'52.8"N, 62°53'52.6"E); Lake Golovka (67°36'06"N, 62°55'28.6"E; 67°35'50"N, 62°55'25.3"E); Lake L (67°35'44.5"N, 62°49'39.2"E), temporary pond near Kharbey (67°58'00"N; 62°34'60"E).

############ Ecology.

In bulk of the studied water bodies, *N. communis* was observed on silted sand, boulders with moss and algal cover, and submerged macrophytes (depth 0.3–1.2 m). In Lake Golovka, it lives on a silted substrate at a depth of 7.5 m. It was also found in small lakes with moss mats floating off shore.

########### 12. Nais elinguis

Taxon classificationAnimaliaTubificidaNaididae

Müller, 1774

F81CEE08-309C-5E55-9DEF-E39B45B433FB

############ Geographic distribution.

Cosmopolitan species. In the Russian tundra: Murmansk Region (Timm and Popchenko 1978), Vaygach Island (Leshko et al 2008), Lake Bolshoy Ngosovey, lakes in the More-yu River basin and the Kara River basin (Baturina and Loskutova 2010), lakes in the Malaya Usa River basin (Baturina et al. 2014b).

############ Location.

Lake Bolshoy Kharbey (67°35'7.7"N, 62°54'46.9"E; 67°34'34.3"N, 62°52'17.4"E; 67°33'48.2"N, 62°55'2.6"E; 67°34'3.5"N, 62°52'17.9"E); Lake K1 (67°36'27.6"N, 62°51'58.4"E); Lake Golovka (67°36'9.4"N, 62°56'39.9"E).

############ Ecology.

Specimens were sampled among pebbles and boulders, as well as on sand or submerged macrophytes (depth 2.5 m or less).

########### 13. Nais pardalis

Taxon classificationAnimaliaTubificidaNaididae

Piguet, 1906

AC2431CE-016B-5736-82AB-B365A8C46F94

############ Geographic distribution.

Holarctic species. In the Russian tundra: Murmansk Region (Timm and Popchenko 1978), the Pechora River delta (Baturina 2018), Vaygach Island (Leshko et al. 2008), the Vashutkiny lakes system (Finogenova 1966), lakes in the Malaya and Bolshaya Usa Rivers basins (Baturina and Loskutova 2010; Baturina et al. 2014b).

############ Location.

Lake Bolshoy Kharbey (67°32'50"N, 62°52'26.3"E; 67°34'N, 62°57'E); temporary near Kharbey (67°58'00"N, 62°34'60"E).

############ Ecology.

The species was recorded along the lakeshore at a depth of 0.8 m or less, on pebble-gravel or silty substrates, and in hollows and small bodies of water, formed by cross-country tracks.

########### 14. Nais pseudobtusa

Taxon classificationAnimaliaTubificidaNaididae

Piguet, 1906

1ECDB743-6176-5E00-90FF-EBF2639CDE06

############ Geographic distribution.

Holarctic species. In the Russian tundra: Murmansk Region (Finogenova 1975), the Pechora River delta (Baturina 2018), the Vashutkiny lakes system (Finogenova 1966), Lake Ambarty and some other lakes in the Korotaikha River basin (Popchenko 1978), lakes in the More-yu River basin, Lake Bolshoy Ngosovey (Baturina and Loskutova 2010), lakes in the Malaya and Bolshaya Usa Rivers basins, as well as lakes in the Kara River basin (Baturina et al. 2014b).

############ Location.

Lake Bolshoy Kharbey (67°34'42.3"N, 62°52'57.1"E; 67°34'34.3"N, 62°52'17.4"E; 67°34'3.5"N, 62°52'17.9"E; 67°33'48.2"N, 62°55'2.6"E; 67°32'50"N, 62°52'26.3"E; 67°32'49.9"N, 62°53'40.1"E); Lake K2 (67°32'55.7"N, 62°51'39.7"E; 67°32'44.5"N, 62°51'38.2"E); Lake D1 (67°35'52.8"N, 62°53'52.6"E), Lake K1 (67°36'27.6"N, 62°51'58.4"E); Lake Golovka (67°36'06"N, 62°55'28.6"E), temporary pond near Kharbey (67°58'00"N, 62°34'60"E).

############ Ecology.

Specimens were sampled from different hard substrates or on the vegetated areas (depth 0.3–2.0 m). In water bodies adjacent to Kharbey, *N. pseudobtusa* prefers clayey or silted sand (depth of 7.5 m); single specimens were found in small isolated lakes with floating moss mats and thick sedge overgrowths.

########### 15. Nais simplex

Taxon classificationAnimaliaTubificidaNaididae

Piguet, 1906

52F106B8-04A9-506B-A737-56BF9AA6DEA4

############ Geographic distribution.

Cosmopolitan species. In the Russian tundra: Murmansk Region (Veselov 1977, Finogenova 1975, Timm and Popchenko 1978), the upper reaches of the Adzva River, (Finogenova 1966), Lake Ambarty and some other lakes in the Korotaikha River basin (Popchenko 1978).

############ Location.

Lake Bolshoy Kharbey (67°31'38"N, 62°53'2.8"E; 67°32'22.1"N, 62°52'10.7"E).

############ Ecology.

The species was found mainly on sand between stones at a depth of 1.0 m or less.

########### 16. Nais variabilis

Taxon classificationAnimaliaTubificidaNaididae

Piguet, 1906

1209FE88-87F0-55D8-A005-27AE70F9E1A0

############ Geographic distribution.

Cosmopolitan species. In the Russian tundra: Murmansk Region (Stalmakova 1974; Finogenova 1975), Lake Vanyuk-ty (Finogenova 1966), Lake Ambarty and some other lakes in the Korotaikha River basin (Popchenko 1978), Lake Bolshoy Ngosovey, lakes in the Kara and More-yu Rivers basins (Baturina and Loskutova 2010), lakes in the Malaya and Bolshaya Usa Rivers basins (Baturina et al. 2014b), Yuribej River floodplain lakes (Zaloznyj 1976), North of Western Siberia (Zaloznyj 1984).

############ Location.

Lake Bolshoy Kharbey (67°34'42.3"N, 62°52'57.1"E; 67°32'44.2"N, 62°55'22.3"E; 67°32'49.4"N, 62°53'6.6"E; 67°31'38"N, 62°53'2.8"E); Lake K1 (67°36'27.6"N, 62°51'58.4"E); Lake Golovka (67°35'50"N, 62°55'25.3"E); temporary pond near Kharbey (67°58'00"N, 62°34'60"E).

############ Ecology.

The species was collected from submerged macrophytes, or stones and sand with moss cover (depth 0.2–1.3 m). It was also found in small water bodies or in humid depressions without open water.

########## Genus *Piguetiella* Sperber, 1939

########### 17. Piguetiella blanci

Taxon classificationAnimaliaTubificidaNaididae

(Piguet, 1906)

3B2E7C6D-144B-5F9F-8EFD-13EECFBFC88A

Nais blanci Piguet, 1906

############ Geographic distribution.

Western Palaearctic, NE of the USA. In the Russian tundra: Murmansk Region (Timm and Popchenko 1978, Finogenova 1975), the Pechora River delta (Baturina 2018), the Vashutkiny lakes system (Finogenova 1966), Lake Ambarty and some other lakes in the Korotaikha River basin (Popchenko 1978), lakes in the Kara River basin (Baturina and Loskutova 2010).

############ Location.

Lake Bolshoy Kharbey (67°34'34.3"N, 62°52'17.4"E; 67°34'N, 62°57'E; 67°34'3.5"N, 62°52'17.9"E; 67°33'48.2"N, 62°55'2.6"E; 67°32'49.9"N, 62°53'40.1"E); Lake L (67°35'44.5"N, 62°49'39.2"E), Lake K2 (67°32'56.1"N, 62°51'39.7"E); Lake K1 (67°36'27.6"N, 62°51'58.4"E; 67°36'17.6"N, 62°52'35"E); Lake Golovka (67°35'50"N, 62°55'25.3"E; 67°36'9.1"N, 62°55'50.6"E).

############ Ecology.

The species inhabits sandy and stony-sandy substrates with algae and moss at depths of 0.5–2.5 m except Lake Bolshoy Kharbey, where it occurs at a depth of 6.2 m.

########## Genus *Ripistes* Dujardin, 1842

########### 18. Ripistes parasita

Taxon classificationAnimaliaTubificidaNaididae

(Schmidt, 1847)

AD2C0416-14F5-5C87-BF9A-F11EACEAD0AF

Stylaria parasita Schmidt, 1847
Ripistes rubra Lastočkin, 1926

############ Geographic distribution.

Holarctic species. In the Russian tundra: Murmansk Region (Veselov 1977; Timm and Popchenko 1978), lakes in the More-yu River basin (Baturina and Loskutova 2010), the Pechora River delta (Baturina 2018), northern part of Western Siberia (Zaloznyj 1984).

############ Location.

Lake Bolshoy Kharbey (67°34'42.3"N, 62°52'57.1"E; 67°34'3.5"N, 62°52'17.9"E; 67°33'45.9"N, 62°54'34.7"E; 67°32'44.2"N, 62°55'22.3"E; 67°32'50"N, 62°52'26.3"E; 67°31'38"N, 62°53'2.8"E); Lake L (67°35'41.5"N, 62°49'34.7"E; 67°35'46"N, 62°49'44.8"E); Lake K2 (67°32'56.1"N, 62°51'39.7"E); Lake K1 (67°36'27.6"N, 62°51'58.4"E; 67°36'22.1"N, 62°52'20.3"E); Lake Golovka (67°35'50"N, 62°55'25.3"E; 67°36'9.1"N, 62°55'50.6"E).

############ Ecology.

The worms were often found in silt, less often on sandy-stony substrate with algae and moss cover, or on submerged macrophytes (depth 0.2–2.5 m, occasionally up to 6.0 m).

########## Genus *Slavina* Vejdovský, 1884

########### 19. Slavina appendiculata

Taxon classificationAnimaliaTubificidaNaididae

(Udekem, 1855)

17981F40-A2DC-5A7C-A75B-F4A4F6E01DAB

Nais appendiculata Udekem, 1855

############ Geographic distribution.

Cosmopolitan species. In the Russian tundra: Murmansk Region (Timm and Abarenkov 2018), the Vashutkiny lakes system (Finogenova 1966) Lake Ambarty and some other lakes in the Korotaikha River basin (Popchenko 1978), lakes in the More-yu River basin (Baturina and Loskutova 2010), lakes in the Kara River basin (Baturina et al. 2014b), the Yamal Peninsula (Stepanov 2017).

############ Location.

Lake Bolshoy Kharbey (67°35'7.7"N, 62°54'46.9"E; 67°34'3.5"N, 62°52'17.9"E; 67°34'N, 62°57'E; 67°32'48.3"N, 62°53'49.7"E; 67°32'22.1"N, 62°52'10.7"E); Lake Golovka (67°35'50"N, 62°55'25.3"E; 67°36'9.1"N, 62°55'50.6"E); Lake K1 (67°36'22.1"N, 62°52'20.3"E; 67°36'17.6"N, 62°52'35"E); temporary pond near Kharbey (67°58'00"N, 62°34'60"E).

############ Ecology.

In the studied lakes, *S. appendiculata* inhabits mainly silt or stones with algal cover (depth 0.2–6.0 m). The worms were less often found in small or temporary ponds with floating moss mats and overgrowths of sedges off the shore.

########## Genus *Specaria* Sperber, 1939

########### 20. Specaria josinae

Taxon classificationAnimaliaTubificidaNaididae

(Vejdovský, 1884)

EEDE360B-50FC-5A86-B04E-0217A8B5C028

Nais josinae Vejdovský, 1884

############ Geographic distribution.

Holarctic species. In the Russian tundra: Murmansk Region (Finogenova 1975; Timm and Popchenko 1978), the Pechora River delta (Baturina 2018), lakes in the More-yu River basin (Baturina and Loskutova 2010), lakes in the Malaya Usa River basin (Baturina et al. 2014b), the Anadyr River basin (Morev 1983b).

############ Location.

Lake Bolshoy Kharbey (67°35'7.7"N, 62°54'46.9"E; 67°34'3.5"N, 62°52'17.9"E; 67°32'44.2"N, 62°55'22.3"E; 67°32'50"N, 62°52'26.3"E; 67°32'49.9"N, 62°53'40.1"E); Lake Golovka (67°36'21.2"N, 62°56'6.6"E); Lake D1 (67°35'52.8"N, 62°53'52.6"E); Lake K1 (67°36'27.6"N, 62°51'58.4"E; 67°36'22.1"N, 62°52'20.3"E); Lake K2 (67°32'56.1"N, 62°51'39.7"E).

############ Ecology.

The species was recorded in most lakes of the study area, mainly on silted sand or clay (depths 0.5–1.1 m), rarely deeper on silted stony-sand or detritus (depths 4.2–6.0 m).

########## Genus *Stylaria* Lamarck, 1816

########### 21. Stylaria fossularis

Taxon classificationAnimaliaTubificidaNaididae

Leidy, 1852

B565BD3B-07B9-5D89-A615-309836CE9BD8

############ Geographic distribution.

North America, Europa, Asia. In the Russian tundra: the Anadyr River basin (Morev 1983b).

############ Location.

Lake Bolshoy Kharbey (67°33'48.2"N, 62°55'2.6"E; 67°32'48.3"N, 62°53'49.7"E); Lake K1 (67°36'27.6"N, 62°51'58.4"E; 67°36'22.1"N, 62°52'20.3"E).

############ Ecology.

The species is rare. It was noted in Lake Bolshoy Kharbey on silted sands (depth 9.5–13.8 m); in adjacent to Kharbey lakes is on clay substrate (depth no more than 0.8 m).

########### 22. Stylaria lacustris

Taxon classificationAnimaliaTubificidaNaididae

(Linnaeus, 1767)

B0ABB032-CFA3-5084-B379-546C6069385F

Nereis lacustris Linnaeus, 1767
Nais proboscidea Müller, 1774

############ Geographic distribution.

Holarctic species. In the Russian tundra: Murmansk Region (Timm and Abarenkov 2018), the Pechora River delta (Baturina 2018), Lake Ambarty and some other lakes in the Korotaikha River basin (Popchenko 1978), the Poluj River basin (Zaloznyj 1976), the Yamal Peninsula (Stepanov 2016, 2017), the Ob River delta (Timm and Abarenkov 2018), northern part of Western Siberia (Zaloznyj 1984), the Anadyr River basin (Morev 1983b).

############ Location.

Lake Bolshoy Kharbey (67°34'34.3"N, 62°52'17.4"E; 67°32'50"N, 62°52'26.3"E); Lake K2 (67°32'55.7"N, 62°51'39.7"E); Lake K1 (67°36'29.4"N, 62°52'58.3"E); Lake Golovka (67°35'50"N, 62°55'25.3"E; 67°36'9.4"N, 62°56'39.9"E).

############ Ecology.

The worms are very mobile; they prefer submerged macrophytes as substrate. *S. lacustris* was also observed on silted stones (depth 0.3–1.0 m).

########## Genus *Uncinais* Levinsen, 1884

########### 23. Uncinais uncinata

Taxon classificationAnimaliaTubificidaNaididae

(Ørsted, 1842)

21FA9388-E51B-53E8-B53D-6D8FA2C9D073

Nais uncinata Ørsted, 1842

############ Geographic distribution.

Holarctic species. In the Russian tundra: Murmansk Region (Finogenova 1975; Veselov 1977; Timm and Popchenko 1978), the Pechora River delta (Baturina 2018), the Vashutkiny lakes system (Finogenova 1966), Lake Ambarty and some other lakes in the Korotaikha River basin (Popchenko 1978), lakes in the Kara River, the More-yu River, and the Bolshaya Usa River basins (Baturina and Loskutova 2010), lakes in the Malaya Usa River basin (Baturina et al. 2014b), the Lena River and Ob River deltas (Gukov 1990), northern part of Western Siberia (Zaloznyj 1984), the Yamal Peninsula (Stepanov 2016), the Anadyr River basin (Morev 1983b).

############ Location.

Lake Bolshoy Kharbey (67°34'34.3"N, 62°52'17.4"E; 67°35'27.5"N, 62°55'30.7"E; 67°34'3.5"N, 62°52'17.9"E; 67°33'48.2"N, 62°55'2.6"E; 67°32'48.3"N, 62°53'49.7"E; 67°32'49.9"N, 62°53'40.1"E); Lake Golovka (67°36'9.4"N, 62°56'39.9"E; 67°36'21.2"N, 62°56'6.6"E); Lake K1 (67°36'17.6"N, 62°52'35"E); Lake K2 (67°32'55.7"N, 62°51'39.7"E).

############ Ecology.

The species was recorded on silt, silted sand, and boulders, from the edge of the water to a depth of 4.5 m. In adjacent to Kharbey lakes, *U. uncinata* prefers moss and algal cover among the rocks in the shore zone. Common species for tundra zone.

########## Genus *Vejdovskyella* Michaelsen, 1903

########### 24. Vejdovskyella comata

Taxon classificationAnimaliaTubificidaNaididae

(Vejdovský, 1884)

AA1A9711-000D-5CAF-8DB8-79B5E73D08E7

Bohemilla comata Vejdovský, 1884

############ Geographic distribution.

Holarctic species. In the Russian tundra: Murmansk Region (Finogenova 1975; Veselov 1977; Timm and Popchenko 1978), the Vashutkiny lakes system (Finogenova 1966), Lake Ambarty and some other lakes in the Korotaikha River basin (Popchenko 1978), lakes in the Kara River and the More-yu River basins, and Lake Bolshoy Ngosovey (Baturina and Loskutova 2010).

############ Location.

Lake Bolshoy Kharbey (67°34'42.3"N, 62°52'57.1"E; 67°32'48.3"N, 62°53'49.7"E; 67°32'49.9"N, 62°53'40.1"E); Lake L (67°35'41.5"N, 62°49'34.7"E); Lake K2 (67°32'55.7"N, 62°51'39.7"E; 67°32'44.5"N, 62°51'38.2"E); Lake K1 (67°36'22.1"N, 62°52'20.3"E); Lake Golovka (67°35'50"N, 62°55'25.3"E; 67°36'17.3"N, 62°56'13.1"E).

############ Ecology.

Within the area of study, *V. comata* was recorded infrequently, living on sand, silt, and clay (depths 4.5–6.5 m, rarer up to 9.8 m).

########### 25. Vejdovskyella macrochaeta

Taxon classificationAnimaliaTubificidaNaididae

(Lastočkin, 1921)

A6A2B4EE-9606-5ACC-BDD9-649C521E8F41

Bohemilla macrochaeta Lastočkin, 1921
Vejdovskyella grandisetosa Finogenova, 1962

############ Geographic distribution.

Eastern Europe. In the Russian tundra: the Pechora River delta (Baturina 2018), the Vashutkiny lakes system (Finogenova 1966), lakes in the Bolshaya Usa River basin (Baturina and Loskutova 2010), lakes in the Malaya Usa River basin (Baturina et al. 2014b).

############ Location.

Lake Bolshoy Kharbey (67°33'45.9"N, 62°54'34.7"E; 67°34'55.7"N, 62°57'44"E); Lake L (67°35'44.5"N, 62°49'39.2"E); Lake K1 (67°36'27.6"N, 62°51'58.4"E); Lake Golovka (67°36'06"N, 62°55'28.6"E; 67°36'9.1"N, 62°55'50.6"E).

############ Ecology.

The species prefers sandy-clay or clay at the shore sites. Rarer, *V. macrochaeta* was observed on clay sediments at a depth of 8.0-9.5 m.

######### Subfamily Pristininae Lastočkin, 1921

########## Genus *Pristina* Ehrenberg, 1828

########### 26. Pristina aequiseta

Taxon classificationAnimaliaTubificidaNaididae

Bourne, 1891

ADDD6166-6550-5851-83F4-7B11C67696DE

Naidium foreli Piguet, 1906

############ Geographic distribution.

Cosmopolitan species. In the Russian tundra: Murmansk Region (Veselov 1977), the Yamal Peninsula (Stepanov 2017).

############ Location.

Lake Bolshoy Kharbey (67°34'42.3"N, 62°52'57.1"E); Lake Golovka (67°35'50"N, 62°55'25.3"E).

############ Ecology.

The species is rare in the area. It was found in the coastal zone of only two lakes, where it was on stones with algae and moss cover, sand or submerged macrophytes.

########### 27. Pristina amphibiotica

Taxon classificationAnimaliaTubificidaNaididae

Lastočkin, 1927

2EA05FB1-3ED6-508E-A64A-7BE94DACE4BD

############ Geographic distribution.

Cosmopolitan. In the Russian tundra: Vaygach Island (Leshko et al. 2008); Lake Ambarty and some other lakes in the Korotaikha River basin (Popchenko 1978).

############ Location.

Lake Bolshoy Kharbey (67°33'48.2"N, 62°55'2.6"E).

############ Ecology.

The species was observed only in the shore zone on the silted sand and pebbles, or on submerged macrophytes.

########### 28. Pristina bilobata

Taxon classificationAnimaliaTubificidaNaididae

(Bretscher, 1903)

0DC2CECB-1F78-5206-983B-DB00E802C5BE

Naidium bilobata Bretscher, 1903

############ Geographic distribution.

Europe. In the Russian tundra: Lake Ambarty and some other lakes in the Korotaikha River basin (Popchenko 1978), lakes in the Malaya Usa River basin (Baturina and Loskutova 2010).

############ Location.

Lake Bolshoy Kharbey (67°32'44.2"N, 62°55'22.3"E); temporary pond near Kharbey (67°58'00"N; 62°34'60"E).

############ Ecology.

The rare species. It inhabits shallow areas with sandy-clayey substrates.

########### 29. Pristina komi

Taxon classificationAnimaliaTubificidaNaididae

Popchenko, 1988

052F1E95-82FE-5723-8EAE-6FC99EE6CB0C

############ Geographic distribution.

European north of Russia (Popchenko 1988).

############ Location.

Lake Bolshoy Kharbey (67°34'42.3"N, 62°52'57.1"E).

############ Ecology.

Several individuals were found on large pebbles with moss and algal cover located in shallow areas.

########### 30. Pristina longiseta

Taxon classificationAnimaliaTubificidaNaididae

Ehrenberg, 1828

5605C5F6-AC66-54D8-BB26-9AB6A323C2F8

############ Geographic distribution.

Cosmopolitan species. In the Russian tundra: Lodejnyj Island (Timm and Abarenkov 2018).

############ Location.

Lake L (67°35'41.5"N, 62°49'34.7"E).

############ Ecology.

Several individuals of *P. longiseta* were found on macrophytes at a depth of 0.5 m.

######### Subfamily Rhyacodrilinae Hrabĕ, 1963

########## Genus *Rhyacodrilus* Bretscher, 1901

########### 31. Rhyacodrilus coccineus

Taxon classificationAnimaliaTubificidaNaididae

(Vejdovský, 1876)

F60937F2-D913-59B1-A32C-D165AAC3589B

Tubifex coccineus Vejdovský, 1876
Tubifex lunzensis Pointner, 1914

############ Geographic distribution.

Holarctic species but recorded also from Australia and Antarctic islands. In the Russian tundra: Murmansk Region (Timm and Popchenko 1978), the Pechora River delta (Baturina 2018), the Vashutkiny lakes system (Finogenova 1966), Lake Ambarty and some other lakes in the Korotaikha River basin (Popchenko 1978), lakes in the Kara River basin (Baturina et al. 2014b), northern part of Western Siberia (Zaloznyj 1984), the Anadyr River basin (Morev 1983a,b), Chukotka Peninsula (Sokolskaya 1986).

############ Location.

Lake Bolshoy Kharbey (67°32'44.2"N, 62°55'22.3"E); temporary pond near Kharbey (67°58'00"N, 62°34'60"E).

############ Ecology.

The species was observed in the shore zone on silted sand between stones. In temporary pond, *R. coccineus* was found in wet moss.

######### Subfamily Tubificinae Vejdovský, 1884

########## Genus *Aulodrilus* Bretscher, 1899

########### 32. Aulodrilus limnobius

Taxon classificationAnimaliaTubificidaNaididae

Bretscher, 1899

A35FDA4D-8429-58C8-BFCC-7F524EC59829

############ Geographic distribution.

Almost cosmopolitan species. In the Russian tundra: Murmansk Region (Timm and Popchenko 1978), the Pechora River delta (Baturina 2018), Lake Bolshoy Ngosovey (Baturina and Loskutova 2010), northern part of Western Siberia (Zaloznyj 1984), the Yenisey River delta (Chekanovskaya 1956), the Lena River delta (Timm and Abarenkov 2018).

############ Location.

Lake Bolshoy Kharbey (67°32'22.1"N, 62°52'10.7"E); Lake L (67°35'41.5"N, 62°49'34.7"E), Lake K2 (67°32'56.1"N, 62°51'39.7"E).

############ Ecology.

Single specimens were observed on silt and clay, at depths up to 5.8 m.

########### 33. Aulodrilus pigueti

Taxon classificationAnimaliaNaididae

Kowalewski, 1914

A7801788-5BE6-5CAF-ABE4-67D43F620EFD

############ Geographic distribution.

Cosmopolitan. In the Russian tundra: lakes in the More-yu River basin (Baturina and Loskutova 2010), Lake Ayan (Zinovjev 1981).

############ Location.

Lake Bolshoy Kharbey (67°34'42.3"N, 62°52'57.1"E).

############ Ecology.

The only specimen was found at a depth of 9.8 m.

########### 34. Aulodrilus pluriseta

Taxon classificationAnimaliaTubificidaNaididae

(Piguet, 1906)

627D1123-4E81-51FC-B7BB-2E329764E4AC

Naidium pluriseta Piguet, 1906

############ Geographic distribution.

Holarctic species, including the Sino-Indian Region and Australia. In the Russian tundra: the Vashutkiny lakes system (Finogenova 1966), Lake Ambarty and some other lakes in the Korotaikha River basin (Popchenko 1978).

############ Location.

Lake Bolshoy Kharbey (67°34'3.5"N, 62°52'17.9"E); Lake K2 (67°32'56.1"N, 62°51'39.7"E).

############ Ecology.

The species lives on slightly silted clay (depth 1.5–5.2 m).

########## Genus *Embolocephalus* Randolph, 1892

########### 35. Embolocephalus velutinus

Taxon classificationAnimaliaTubificidaNaididae

(Grube, 1879)

FFBCFECA-0362-5102-858A-FD4652240288

Saenuris velutinus Grube, 1879
Tubifex sarnensis Pierantoni, 1904
Peloscolex fontinalis Hrabĕ, 1964

############ Geographic distribution.

Central and Southern Europe, possibly also northern Europe and Siberia (Timm 2009). In the Russian tundra: the Vashutkiny lakes system (Finogenova 1966), Lake Ambarty and some other lakes in the Korotaikha River basin (Popchenko 1978), the Yamal Peninsula (Stepanov 2016).

############ Location.

Lake Bolshoy Kharbey (67°34'34.3"N, 62°52'17.4"E; 67°32'50"N, 62°52'26.3"E; 67°32'49.9"N, 62°53'40.1"E); Lake Golovka (67°36'9.4"N, 62°56'39.9"E).

############ Ecology.

The species is common in the tundra zone. It lives on various sediments (including rocky, sandy, clayey, or silty) in the littoral zone of lakes.

########## Genus *Ilyodrilus* Eisen, 1879

########### 36. Ilyodrilus templetoni

Taxon classificationAnimaliaTubificidaNaididae

(Southern, 1909)

D7AE0D95-706B-55B7-B2B4-6F9B5ECD35D3

Tubifex templetoni Southern, 1909

############ Geographic distribution.

Holarctic species. In the Russian tundra: Lake Ambarty and some other lakes in the Korotaikha River basin (Popchenko 1978).

############ Location.

Lake Bolshoy Kharbey (67°34'3.5"N, 62°52'17.9"E); Lake Golovka (67°36'17.3"N, 62°56'13.1"E).

############ Ecology.

The species prefers soft silts of profundal zone of the lakes (depth of 6.0–6.5 m).

########## Genus *Isochaetides* Hrabĕ, 1966

########### 37. Isochaetides michaelseni

Taxon classificationAnimaliaTubificidaNaididae

(Lastočkin, 1936)

5B69F49E-1C3C-5367-9A88-ABA122EFA9D5

Limnodrilus michaelseni Lastočkin, 1936

############ Geographic distribution.

Inhabits Eastern Europe. In the Russian tundra: Lake Ambarty and some other lakes in the Korotaikha River basin (Popchenko 1978).

############ Location.

Lake Golovka (67°36'21.2"N, 62°56'6.6"E).

############ Ecology.

This species is rare in the tundra lakes. It was found on clayey substrate at a depth of 1.7 m.

########## Genus *Limnodrilus* Claparède, 1862

########### 38. Limnodrilus hoffmeisteri

Taxon classificationAnimaliaTubificidaNaididae

Claparède, 1862

E8256212-7381-5FBC-ABED-F9B2D7EAC190

Limnodrilus parvus Southern, 1909

############ Geographic distribution.

Cosmopolitan species. In the Russian tundra: Murmansk Region (Stalmakova 1974; Veselov 1977; Timm and Popchenko 1978; Jakovlev 1982); the Pechora River delta (Baturina 2018), Vaygach Island (Leshko et al. 2008), Lake Ambarty and some other lakes in the Korotaikha River basin (Popchenko 1978), lakes in the More-yu River basin (Baturina and Loskutova 2010), the Norilsk`s group of lakes (Vershinin 1960), the Ob River delta (Timm and Abarenkov 2018), northern part of Western Siberia (Zaloznyj 1984), the Gydansky and Yamal Peninsulas (Stepanov 2016, 2017, 2018), the Lena River delta (Gukov 1990).

############ Location.

Lake Bolshoy Kharbey (67°34'55.7"N, 62°57'44"E; 67°33'48.2"N, 62°55'2.6"E; 67°32'44.2"N, 62°55'22.3"E; 67°32'48.3"N, 62°53'49.7"E); Lake L (67°35'41.5"N, 62°49'34.7"E); Lake K2 (67°32'56.1"N, 62°51'39.7"E); Lake K1 (67°36'27.6"N, 62°51'58.4"E); Lake Golovka (67°36'9.4"N, 62°56'39.9"E; 67°36'9.1"N, 62°55'50.6"E).

############ Ecology.

The species is recorded in most lakes of the Kharbey system. *Limnodrilus hoffmeisteri* prefers silts, but it was also found on sandy-clay sediment and stones with algal cover (from the water edge to 5.2 m in depth).

########### 39. Limnodrilus udekemianus

Taxon classificationAnimaliaTubificidaNaididae

Claparède, 1862

F9AC6156-267B-5BF5-86A3-09D6CFF56C96

Isochaeta virulenta Pointner, 1911

############ Geographic distribution.

Cosmopolitan species. In the Russian tundra: Murmansk Region (Stalmakova 1969; Veselov 1977; Timm and Popchenko 1978), the Vashutkiny lakes system (Finogenova 1966), Lake Ambarty and some other lakes in the Korotaikha River basin (Popchenko 1978), lakes in the More-yu River basin (Baturina and Loskutova 2010), northern part of Western Siberia and the Yamal Peninsula (Zaloznyj 1984).

############ Location.

Lake Bolshoy Kharbey (67°33'48.2"N, 62°55'2.6"E; 67°31'38"N, 62°53'2.8"E; 67°32'49.9"N, 62°53'40.1"E).

############ Ecology.

The species was found on stones with moss or algal cover and clayey ground (depth 1.1–2.0 m). It was not widely distributed in the Kharbey lakes system previously.

########## Genus *Potamothrix* Vejdovský & Mrázek, 1903

########### 40. Potamothrix hammoniensis

Taxon classificationAnimaliaTubificidaNaididae

(Michaelsen, 1901)

147A5ED8-D9AA-5F99-83FC-5F8A4AA4734C

Ilyodrilus hammoniensis Michaelsen, 1901
Tubifex cameranoi De Visart, 1901
Psammoryctes fossor Ditlevsen, 1904

############ Geographic distribution.

It was found in Western Palearctic, Africa, Great Lakes of North America, and Lake Titicaca in South America. In the Russian tundra: Murmansk Region (Stalmakova 1974), the Pechora River delta (Baturina 2018), lakes in the More-yu River basin (Baturina and Loskutova 2010), the Ob River and Lena River deltas (Timm and Abarenkov 2018), the Yamal Peninsula (Stepanov 2018).

############ Location.

Lake Bolshoy Kharbey (67°34'3.5"N, 62°52'17.9"E; 67°31'38"N, 62°53'2.8"E); Lake Golovka (67°36'21.2"N, 62°56'6.6"E).

############ Ecology.

The species was recorded on silty, clayey, sandy-silty sediments, submerged macrophytes or algal cover (depth 0.4–1.1 m).

########## Genus *Spirosperma* Eisen, 1879

########### 41. Spirosperma ferox

Taxon classificationAnimaliaTubificidaNaididae

Eisen, 1879

4F5D8746-9AA7-5EBD-A005-5FDD79379E9D

Peloscolex ferox (Eisen, 1879)
Embolocephalus plicatus Randolph, 1892

############ Geographic distribution.

Holarctic species. In the Russian tundra: the Pechora River delta (Baturina 2018), Lake Ambarty and some other lakes in the Korotaikha River basin (Popchenko 1978), lakes of central part of Bolshezemelskaya tundra (Belyakov and Skvortsov 1994), lakes in the Malaya and Bolshaya Usa Rivers basins, as well as lakes in the Kara River basin (Baturina et al. 2014b), lakes in the More-yu River basin (Baturina and Loskutova 2010), the Norilsk group of lakes (Vershinin 1960), northern part of Western Siberia (Zaloznyj 1984), the Yamal Peninsula (Stepanov 2016, 2017, 2018).

############ Location.

Lake Bolshoy Kharbey (67°34'34.3"N, 62°52'17.4"E; 67°35'27.5"N, 62°55'30.7"E; 67°34'3.5"N, 62°52'17.9"E; 67°33'48.2"N, 62°55'2.6"E; 67°32'44.2"N, 62°55'22.3"E; 67°32'50"N, 62°52'26.3"E; 67°31'38"N, 62°53'2.8"E; 67°32'22.1"N, 62°52'10.7"E); Lake Golovka (67°35'50"N, 62°55'25.3"E; 67°36'9.1"N, 62°55'50.6"E; 67°36'17.3"N, 62°56'13.1"E); Lake D1 (67°35'52.8"N, 62°53'52.6"E); Lake K1 (67°36'27.6"N, 62°51'58.4"E) Lake K2 (67°32'55.7"N, 62°51'39.7"E); Lake L (67°35'41.5"N, 62°49'34.7"E; 67°35'46"N, 62°49'44.8"E); temporary pond (67°58' N; 62°34'60"E).

############ Ecology.

This species is widespread in the area; it was found in most studied water bodies. It inhabits various grounds: stony, sandy, and muddy, often occurs on stones covered by moss or algae or submerged macrophytes (from the water edge up to 9.0 m). In most lakes, *S. ferox* is dominant in number.

########## Genus *Lophochaeta* Štolc, 1886

########### 42. Lophochaeta ignota

Taxon classificationAnimaliaTubificidaNaididae

(Štolc, 1886)

53D4A6CE-2C31-5AC6-9A7F-610709BEDE47

Tubifex ignotus (Štolc, 1886)
Tubifex filum Michaelsen, 1901

############ Geographic distribution.

Palearctic species, which was also indicated for Great Lakes of North America and Lake Titicaca in South America. In the Russian tundra: Murmansk Region (Finogenova 1975; Timm and Popchenko 1978), the Pechora River delta (Baturina 2018), Lake Ambarty and some other lakes in the Korotaikha River basin (Popchenko 1978), lakes in the More-yu River Kara River basins (Baturina and Loskutova 2010), lakes in the Malaya Usa River basin (Baturina et al. 2014b).

############ Location.

Lake Bolshoy Kharbey (67°34'42.3"N, 62°52'57.1"E; 67°35'7.7"N, 62°54'46.9"E; 67°34'3.5"N, 62°52'17.9"E; 67°33'45.9"N, 62°54'34.7"E; 67°32'48.3"N, 62°53'49.7"E), Lake L (67°35'46"N, 62°49'44.8"E); Lake K2 (67°32'55.7"N, 62°51'39.7"E), Lake K1 (67°36'22.1"N, 62°52'20.3"E; 67°36'17.6"N, 62°52'35"E); Lake Golovka (67°36'21.2"N, 62°56'6.6"E; 67°36'06"N, 62°55'28.6"E).

############ Ecology.

The species lives in silt, clay and sand (from the water edge up to 9.5 m in depth).

########## Genus *Tubifex* Lamarck, 1816

########### 43. Tubifex tubifex

Taxon classificationAnimaliaTubificidaNaididae

(Müller, 1774)

5EB8C1B5-47C4-552E-BD9F-6FBF104B5E1C

Lumbricus tubifex Müller, 1774

############ Geographic distribution.

Cosmopolitan species excluding the tropic areas. In the Russian tundra: Murmansk Region (Finogenova 1975; Timm and Popchenko 1978), Vaygach Island (Leshko et al. 2008), the Pechora River delta (Baturina 2018); the Vashutkiny lakes system (Finogenova 1966); Lake Ambarty and some other lakes in the Korotaikha River basin (Popchenko 1978), lakes in central part of Bolshezemelskaya tundra (Belyakov and Skvortsov 1994), lakes in the More-yu River basin, Lake Bolshoy Ngosovey and lakes in the More-yu River basin (Baturina and Loskutova 2010), lakes in the Malaya Usa River basin (Baturina et al. 2014b), the Kara River basin (Baturina and Loskutova 2010), the Norilsk group of lakes (Vershinin 1960), northern part of Western Siberia (Zaloznyj 1984), the Lena River Delta (Gukov 1990), the Gyda River basin (Zaloznyj 1984), the Yamal Peninsula (Stepanov 2016, 2017, 2018), the Tanama River (Gundrizer et al. 1977), and the Yenisey River delta (Chekanovskaya 1956).

############ Location.

Bolshoy Kharbey Lake (67°34'42.3"N, 62°52'57.1"E; 67°34'34.3"N, 62°52'17.4"E; 67°35'7.7"N, 62°54'46.9"E; 67°34'3.5"N, 62°52'17.9"E; 67°33'48.2"N, 62°55'2.6"E; 67°32'48.3"N, 62°53'49.7"E; 67°31'38"N, 62°53'2.8"E); Lake Golovka (67°36'06"N, 62°55'28.6"E; 67°35'50"N, 62°55'25.3"E; 67°36'21.2"N, 62°56'6.6"E); Lake D2 (67°35'52.8"N, 62°53'52.6"E); Lake K1 (67°36'22.1"N, 62°52'20.3"E; 67°36'17.6"N, 62°52'35"E); Lake K2 (67°32'56.1"N, 62°51'39.7"E); Lake L (67°35'41.5"N, 62°49'34.7"E; 67°35'46"N, 62°49'44.8"E); temporary pond (67°58'00"N, 62°34'60"E).

############ Ecology.

This is one of the numerous and widespread oligochaete species in the Kharbey lakes system. It was found on various substrates (from the water edge to 8.0 m in depth).

######### Subfamily Telmatodrilinae Eisen, 1879

########## Genus *Alexandrovia* Hrabĕ, 1962

########### 44. Alexandrovia ringulata

Taxon classificationAnimaliaTubificidaNaididae

(Sokolskaja, 1961)

3E949D94-9D4B-521E-9639-638F5DC3EFEE

Peloscolex ringulatus Sokolskaja, 1961
Alexandrovia onegensis Hrabĕ, 1962

############ Geographic distribution.

Palaearctic species inhabits Lakes of Karelia and Siberia. In tundra zone of Russia: Lake Ambarty and some other lakes in the Korotaikha River basin (Popchenko 1978), lakes in the Kara River basin (Baturina and Loskutova 2010), lakes in the Malaya Usa River basin (Baturina et al. 2014b), the Tanama River (Gundrizer et al. 1978), northern part of Western Siberia (Zaloznyj 1984), the Gyda River basin (Zaloznyj 1976), Lake Taymyr (Timm 1996), the Anadyr River basin (Morev 1983b), and the Chukotka Peninsula (Sokolskaya 1972).

############ Location.

Lake K1 (67°36'17.6"N, 62°52'35"E); Lake Golovka (67°35'50"N, 62°55'25.3"E).

############ Ecology.

Single specimens were washed out from moss cover of sandy-silty substrates or submerged macrophytes.

######## Family Enchytraeidae Vejdovský, 1879

######### Genus *Cognettia* Nielsen et Christensen, 1959

########## 45. Cognettia glandulosa

Taxon classificationAnimaliaTubificidaEnchytraeidae

(Michaelsen, 1888)

25F0049F-E1FD-5AB1-9A6F-2E1158616B19

Pachydrilus glandulosus Michaelsen, 1888
Chamaedrilus glandulosus (Michaelsen, 1888)

########### Geographic distribution.

Holarctic species. In the Russian tundra: the Pechora River delta (Baturina 2018), Lake Ambarty and some other lakes in the Korotaikha River basin (Popchenko 1978), lakes in the More-yu River basin (Baturina and Loskutova 2010), lakes in the Malaya Usa River basin (Baturina et al. 2014b), the Taymyr Peninsula (Nurminen 1980), and the Chukotka Peninsula (Timm and Abarenkov 2018).

########### Location.

Temporary ponds of the Kharbey system (67°58'00"N, 62°34'60"E).

########### Ecology.

The worms were found in ponds that do not have an open water surface, in moss covering the swampy substrate.

########## 46. Cognettia sphagnetorum

Taxon classificationAnimaliaTubificidaEnchytraeidae

(Vejdovský, 1878)

9EA335E1-E258-514F-BC7F-4C1AECB7A8E8

Pachydrilus sphagnetorum Vejdovský, 1878
Chamaedrilus sphagnetorum (Vejdovský, 1878)

########### Geographic distribution.

Previously it was registered only in Europe, eastern part of North America, and Greenland.

########### Location.

Temporary pond near Kharbey (67°58'00"N, 62°34'60"E).

########### Ecology.

The species inhabits pond, which does not have an open water surface, in the moss covering the swamped substrate.

######### Genus *Mesenchytraeus* Eisen, 1878

########## 47. Mesenchytraeus armatus

Taxon classificationAnimaliaTubificidaEnchytraeidae

(Levinsen, 1884)

458B9D3D-FEBA-5153-B4D6-DD0C5FDF950C

Analycus armatus Levinsen, 1884

########### Geographic distribution.

Holarctic species. In tundra zone of Russia: Murmansk Region (Timm and Popchenko 1978), lakes in the Kara River basin (Baturina and Loskutova 2010).

########### Location.

Lake Bolshoy Kharbey (67°34'55.7"N, 62°57'44"E; 67°34'3.5"N, 62°52'17.9"E; 67°32'50"N, 62°52'26.3"E; 67°32'49.9"N, 62°53'40.1"E); Lake K1 (67°36'17.6"N, 62°52'35"E) temporary pond (67°58'00"N, 62°34'60"E).

########### Ecology.

The species was encountered mainly on sandy and sandy-gravel substrate with moss cover in the shore zone. Single specimens were found on the boggy parts of the small lake.

####### Order Lumbriculida Brinkhurst, 1971

######## Family Lumbriculidae Vejdovský, 1884

######### Genus *Lumbriculus* Grube, 1844

########## 48. Lumbriculus alexandrovi

Taxon classificationAnimaliaLumbriculidaLumbriculidae

Popchenko, 1976

AF700553-7853-5A48-9B8D-4B10D72DE737

########### Geographic distribution.

It was known only in the Karelia (NW Russia). In tundra zone of Russia: Lake Ambarty and some other lakes in the Korotaikha River basin (Popchenko 1978).

########### Location.

Lake Golovka (67°36'21.2"N, 62°56'6.6"E).

########### Ecology.

Single specimens were found on silted gravel in shore zone of the lake.

########## 49. Lumbriculus variegatus

Taxon classificationAnimaliaLumbriculidaLumbriculidae

(Müler, 1774)

99E9CF93-F260-5DFA-AA34-757916F061ED

Lumbricus variegatus Müller, 1774
Lumbriculus kareliensis Popchenko, 1976

########### Geographic distribution.

Holarctic species. In tundra zone of Russia: Murmansk Region (Stalmakova 1974; Finogenova 1975; Veselov 1977; Timm and Popchenko 1978), Vaygach Island (Leshko et al 2008), the Pechora River delta (Baturina 2018), Lake Ambarty and some other lakes in the Korotaikha River basin (Popchenko 1978), lakes in the Kara River, Malaya and Bolshaya Usa Rivers basins (Baturina et al. 2014b), the Vashutkiny lakes system (Finogenova 1966), Lake Bolshoy Ngosovey and lakes in the More-yu River basin (Baturina and Loskutova 2010), the Gydansky and Yamal Peninsula (Stepanov 2016, 2017, 2018), northern part of Western Siberia (Zaloznyj 1984), the Yenisey River delta (Chekanovskaya 1956), and the Anadyr River basin (Morev 1983b).

########### Location.

Lake Bolshoy Kharbey (67°34'N, 62°57'E; 67°34'34.3"N, 62°52'17.4"E; 67°34'3.5"N, 62°52'17.9"E; 67°32'44.2"N, 62°55'22.3"E; 67°32'50"N, 62°52'26.3"E; 67°31'38"N, 62°53'2.8"E); Lake D1 (67°36'2.2"N, 62°54'8.2"E; 67°35'52.8"N, 62°53'52.6"E); Lake L (67°35'41.5"N, 62°49'34.7"E); Lake K2 (67°32'40.9"N, 62°51'39.1"E); Lake Golovka (67°36'21.2"N, 62°56'6.6"E, 67°36'9.1"N, 62°55'50.6"E); temporary pond (67°58"N, 62°34'60"E).

########### Ecology.

The species was observed in most lakes of the area; *L. variegatus* often numerically dominated, inhabiting sands or silts between stones, submerged macrophytes, and stones with moss or algal covering (at depth up to 1 m).

######### Genus *Stylodrilus* Claparède, 1862

########## 50. Stylodrilus heringianus

Taxon classificationAnimaliaLumbriculidaLumbriculidae

Claparède, 1862

4B395145-ECD4-5080-B1A1-4A3F27895033

########### Geographic distribution.

Holarctic species. In tundra zone of Russia: Murmansk Region (Timm and Abarenkov 2018), Kara River basin (Baturina and Loskutova 2010), Lake Yurto (Finogenova 1966), the Anadyr River basin (Morev 1983b), lakes in the central part of Bolshezemelskaya tundra (Belyakov and Skvortsov 1994), the Yamal Peninsula (Stepanov 2016, 2017).

########### Location.

Lake Bolshoy Kharbey (67°34'34.3"N, 62°52'17.4"E; 67°35'27.5"N, 62°55'30.7"E); Lake Golovka (67°36'9.4"N, 62°56'39.9"E).

########### Ecology.

This species is in the area. It lives on stones with algal covering, silty or sandy substrates (depth up to 1.5 m).

######## Family Lumbricidae Rafinesque, 1815

######### Genus *Eiseniella* Michaelsen, 1900

########## 51. Eiseniella tetraedra

Taxon classificationAnimaliaLumbriculidaLumbricidae

(Savigny, 1826)

813F7D01-C3FC-5ED5-B02D-D7C7053CB756

Enterion tetraedra Savigny, 1826

########### Geographic distribution.

Western part of the Palearctic Region. In tundra zone of Russia: the Pechora River delta (Baturina 2018), lakes Pervoe Bobrovoe and Akulkino (Finogenova 1975).

########### Location.

Temporary pond (67°58'00"N, 62°34'60"E).

########### Ecology.

The species was found on a swampy substrate with moss cover.

###### Subclass Acanthobdellea Livanow, 1905

####### Order Acanthobdellida Grube, 1851

######## Family Acanthobdellidae Grube, 1851

######### Genus *Acanthobdella* Grube, 1851

########## 52. Acanthobdella peledina

Taxon classificationAnimaliaAcanthobdellidaAcanthobdellidae

Grube, 1851

ED449786-40DB-5DC1-BD66-416F895792DE

########### Geographic distribution.

Palaearctic region, namely Northern Eurasia. In the Russian tundra: the Vashutkiny lakes system (Lukin 1966), northern part of Western Siberia (Zaloznyj 1984), the Gydansky Peninsula (Gagnon and Shorthouse 2019).

########### Location.

Lake Bolshoy Kharbey (67°31'8"N, 62°53'2.8"E), temporary pond (67°58'00"N, 62°34'60"E).

########### Ecology.

Parasite of arctic salmonid fish. Within the area, *A. peledina* was observed on *Coregonus lavaretus* (Linnaeus, 1758) and *Thymallus thymallus* (Linnaeus, 1758).

###### Subclass Hirudinea Lamarck, 1818 (synonym Hirudinida)

####### Order Rhynchobdellida Blanchard, 1894

######## Family Glossiphoniidae Vaillant, 1890

######### Subfamily Glossiphoniinae Vaillant, 1890

########## Genus *Glossiphonia* Johnson, 1817

########### 53. Glossiphonia complanata

Taxon classificationAnimaliaRhynchobdellidaGlossiphoniidae

(Linnaeus, 1758)

B51DEFDE-1A7A-5079-8165-D02B726CC8D3

Hirudo complanata Linnaeus, 1758
Glossiphonia tuberculate Johnson, 1816
Glossiphonia complanata Blanchard, 1894

############ Geographic distribution.

Palaearctic region. Previously mentioned as Holarctic species. However, recent molecular studies confuted its findings in North America (Williams et al. 2013; Kaygorodova et al. 2019). In tundra zone of Russia: some lakes in the Korotaikha River basin (Zaloznyj 1978), the Yamal Peninsula (Stepanov 2016, 2017).

############ Location.

Small nameless lakes near Syattey-ty (67°33'46.2"N, 62°41'32.8"E; 67°33'11.6"N, 62°46'5.4"E; 67°33'13.6"N, 62°42'50.8"E; 67°32'27.9"N, 62°43'40.9"E); Lake D1 (67°36'2.2"N, 62°54'8.2"E; 67°35'52.8"N, 62°53'52.6"E).

############ Ecology.

This eurytopic species is numerically dominant in stagnant waters. Samples were collected from the shore; leeches were found in a free-living state on aquatic vegetation or on the underside of stones.

########### 54. Glossiphonia verrucata

Taxon classificationAnimaliaRhynchobdellidaGlossiphoniidae

(Müller, 1844)

DD68EA04-EB38-5678-A446-F6B15B57FE63

Clepsine verrucata Müller, 1844
Glossiphonia verrucata Johansson, 1909
Batracobdella verrucata Pawlowski, 1936
Boreobdella verrucata Lukin, 1956

############ Geographic distribution.

Palearctic region. In tundra zone of Russia: Lukin reported this species as *Boreobdella verrucata* from Lake Plesovka in the Komi region (Lukin 1956).

############ Location.

No specimen in our collection.

############ Ecology.

The boreal species inhabits the North Eurasia (Lukin 1976; Kaygorodova et al. 2019), including recent findings in The Netherlands (Soes and Cuppen 2004) and France (Nesemann 1990; d'Hondt and Ben Ahmed 2009).

########### 55. Glossiphonia concolor

Taxon classificationAnimaliaRhynchobdellidaGlossiphoniidae

(Apathy, 1888)

41935458-193C-53C3-8B5E-96F43E9F7FEC

Clepsine concolor Apathy, 1888
Glossiphonia concolor Livanow, 1903

############ Geographic distribution.

Palearctic region. In tundra zone of Russia: the Usa River basin (Lukin 1976) and the Pechora River (Zaloznyj 1978).

############ Location.

Temporary pond near the Lake Kharbey (67°58'00"N, 62°34'60"E), Lake Syattey-ty (67°33'13.6"N, 62°42'50.8"E) and small nameless lakes in its neighbourhood (67°33'46.2"N, 62°41'32.8"E; 67°33'11.6"N, 62°46'5.4"E; 67°32'56.7"N, 62°45'58.4"E).

############ Ecology.

This species is known as predator of small molluscs. The leeches were found on swamped places, as well as in small lakes with silted sand substrate.

########## Genus *Hemiclepsis* Vejdovský, 1884

########### 56. Hemiclepsis marginata

Taxon classificationAnimaliaRhynchobdellidaGlossiphoniidae

(Müller, 1774)

44046196-8004-5993-8C96-B656B1B89ECA

Hirudo marginata O. F. Müller, 1774
Piscicola marginata Moquin-Tandon, 1827
Clepsine marginata F. Müller, 1844
Hemiclepsis marginata Harding, 1910

############ Geographic distribution.

Palaearctic region. In tundra zone of Russia: the basin of the Usa (Lukin 1962).

############ Location.

The species was not found in the area.

############ Ecology.

This leech parasitises molluscs, amphibians, and fishes; *H. marginata* is seemingly very rare species in the Komi region. Lukin (1962) has occasionally found one specimen in the Usa River.

######### Subfamily Haementeriinae Autrum, 1939

########## Genus *Helobdella* Blanchard, 1876

########### 57. Helobdella stagnalis

Taxon classificationAnimaliaRhynchobdellidaGlossiphoniidae

(Linnaeus, 1758)

E1EE7967-F72C-5712-9675-0F3C450DFB4A

Hirudo stagnalis Linnaeus 1758
Glossiphonia stagnalis Blanchard 1894
Glossiphonia (Helobdella) stagnalis Moore 1922
Bakedebdella gibbosa Sciacchitiano 1939

############ Geographic distribution.

Cosmopolitan species. In tundra zone of Russia: lakes of Korotaikha River basin (Zaloznyj 1978), the Yamal Peninsula (Stepanov 2016).

############ Location.

The species was not recorded in the Kharbey lakes. It was only found in an oxbow lake in the Pechora River delta (68°8'8.2"N, 53°36'33.8"E).

############ Ecology.

Sandy-clay sediment (depth 0.5 m).

######### Subfamily Theromyzinae Sawyer, 1986

########## Genus *Theromyzon* Philippi, 1867

########### 58. Theromyzon tessulatum

Taxon classificationAnimaliaRhynchobdellidaGlossiphoniidae

(Müller, 1774)

1A280FF0-E0BE-582D-91D9-87A0A19DE034

Protoclepsis tesselata Livanow 1902

############ Geographic distribution.

Holarctic species. In tundra zone of Russia: no data.

############ Location.

Small lakes near Syattey-ty (67°33'46.2"N, 62°41'32.8"E); temporary pond near Kharbey (67°58'00"N, 62°34'60"E).

############ Ecology.

The leeches were found in the moss cover of the substrate in two swamped lakes, which do not have an open water surface.

########### 59. Theromyzon maculosum

Taxon classificationAnimaliaRhynchobdellidaGlossiphoniidae

(Rathke, 1862)

443F6187-FA3A-50D1-B325-8BBE3DF31775

Clepsine maculosa Rathke, 1862
Clepsine maculosa Grube, 1871
Glossiphonia maculosa Vaillant, 1890
Protoclepsine sexoculata Moore, 1898
Protoclepsis meyeri Livanow, 1902
Protoclepsis garjaewi Livanow, 1902
Theromyzon sexoculata Johansson, 1909
Theromyzon maculosa Pawłowski 1936

############ Geographic distribution.

Palearctic region. In tundra zone of Russia: it was reported as *P. maculosum* from Komi Republic region (Lukin 1957, 1962).

############ Location.

No specimen in our collection.

############ Ecology.

This leech normally lives in s a temperate or even relatively cold climate; prefers stagnant freshwater; it parasitises waterfowl, mainly ducks and geese.

######## Family Piscicolidae Johnston, 1865 (synonym Ichthyobdellidae Leuckart, 1863)

######### Genus *Piscicola* Blainville, 1818

########## 60. Piscicola geometra

Taxon classificationAnimaliaRhynchobdellidaPiscicolidae

(Linnaeus, 1761)

7C5CEC30-2C34-5740-8C19-077C62B0CA57

Hirudo geometra Linnaeus, 1758

########### Geographic distribution.

Transpalearctic species. In tundra zone of Russia: Kharbey lakes (Zaloznyj 1978), northern part of Western Siberia (Zaloznyj 1984), the Yamal Peninsula (Stepanov 2016, 2017).

########### Location.

There are findings in Lake Sudorma (67°17'31.07"N, 50°16'25.58"E) situated in the neighbouring area to Bolshezemelskaya tundra.

########### Ecology.

*Piscicola geometra* is considered to be an oxyphilic species. It inhabits both rivers and stagnant water bodies with a favorable oxygen regime. This is an ectoparasite predominantly of cyprinids, with no obvious host preference. A single specimen was sampled from the dorsal fin of a whitefish.

########## 61. Piscicola

Taxon classificationAnimaliaRhynchobdellidaPiscicolidae

sp.

963FE17C-02D1-5D13-8324-A5F855A2270F

########### Morphology.

Very small leech, its body length is 7 mm and diameter is 1.5 mm. Pigmentation is uniform, does not form a specific pattern on the dorsal side of the body, unlike the widespread *P. geometra*.

########### Location.

Lake Golovka (67°35'50"N, 62°55'25.3"E).

########### Ecology.

A single specimen was found on the dorsal fin of a whitefish.

######### Genus *Cystobranchus* Diesing, 1859

########## 62. Cystobranchus mammillatus

Taxon classificationAnimaliaRhynchobdellidaPiscicolidae

(Malm, 1863)

03EE0D1E-B88A-5273-B449-CF9EBC5702D1

Caliobdella mammilata Nesemann, 1994

########### Geographic distribution.

Palaearctic region. In tundra zone of Russia: northern part of Western Siberia (Zaloznyj 1984).

########### Location.

No specimen in our collection from the Kharbey area.

########### Ecology.

A specific parasite of burbot.

###### Order Arhynchobdellida Blanchard, 1894

####### Suborder Erpobdelliformes Sawyer, 1986

######## Family Erpobdellidae Blanchard, 1894

######### Genus *Erpobdella* de Blainville, 1818

########## 63. Erpobdella octoculata

Taxon classificationAnimaliaArhynchobdellidaErpobdellidae

(Linnaeus, 1758)

6AA7F9BD-AA97-5BC6-ABE3-F0413A5EB714

Hirudo octoculata Linnaeus, 1758
Herpobdella octoculata Johansson 1910
Herpobdella octomaculata Pawlowski 1935

########### Geographic distribution.

Palaearctic and Nearctic regions. In tundra zone of Russia: Kharbey lakes and other lakes of the Bolshezemelskaya tundra (Lukin 1956; Zaloznyj 1978), northern part of Western Siberia (Zaloznyj 1984), the Yamal Peninsula (Stepanov 2016).

########### Location.

No specimen in our collection from the Kharbey area. Earlier records were probably misidentified *Erpobdella* sp. 1 (see below).

########### Ecology.

This leech inhabits various types of water bodies; it is considered the most numerous *Erpoddella* in most Palearctic freshwater bodies. These leeches avoid of humic substances, and practically do not occur in distrophic waters. However, *E. octoculata* can inhabit highly polluted water bodies.

########## 64. Erpobdella monostriata

Taxon classificationAnimaliaArhynchobdellidaErpobdellidae

(Lindenfeld & Pietruszynski, 1890)

0618BCC0-F6A6-5CCE-B26E-65D4168ECA65

Nephelis octoculata var. monostriata Lindenfeld & Pietruszynski, 1890
Erpobdella vilnensis (Liskiewitz, 1925) in part

########### Geographic distribution.

Widespread in the Palaearctic region and occurs from the Netherland (van Haaren et al. 2004) in the west to the Voronezh region of Russia in the east (Utevsky et al. 2015). In tundra zone of Russia: this *Erpobdella* is relatively low in numbers in the northwestern European part of Russia: basin of the Northern Dvina, Vychegda and Usa rivers where, *E. nigricollis* dominates according to Lukin (1957).

########### Location.

Lake Bolshoy Kharbey (67°32'48.3"N, 62°53'49.7"E; 67°34'34.3"N; 62°52'17.4"E), Lake D1 (67°36'2.2"N, 62°54'8.2"E; 67°35'52.8"N, 62°53'52.6"E).

########### Ecology.

The species was found in *Arctophila* thickets in a small body of water, and in silt of Lake Kharbey.

########## 65. Erpobdella nigricollis

Taxon classificationAnimaliaArhynchobdellidaErpobdellidae

(Brandes, 1900)

D8179C4B-83FB-51D2-B2A4-DAEEA61CA8ED

Nephelis testacea f. nigricollis Brandes, 1900
Herpobdella testacea var. nigricollis Johansson, 1929

########### Geographic distribution.

Palaearctic region. The *E. nigricollis* geographic range is in the northern part of Eurasia with the Yenisei River as the eastern border. In the tundra zone of Russia, according to Lukin (1976), this leech is most widespread and numerous in water bodies of the Komi region and the eastern part of Arkhangelsk Region.

########### Location.

There is no specimen in our collection from the Kharbey area.

########### Ecology.

According to Nesemann and Neubert (1999), *E. nigricollis* belongs to the potamal fauna and prefers large rivers; in contrast, Lukin (1976) asserts that this leech is typical for small lakes and natural stagnant water bodies located in the floodplain of rivers.

########## 66. Erpobdella testacea

Taxon classificationAnimaliaArhynchobdellidaErpobdellidae

(Savigny, 1822)

9392436B-03DE-5AB2-BD5A-56A06A1838F8

Nephelis testacea Savigny, 1822
Herpobdella testacea Blanchard, 1894

########### Geographic distribution.

Palaearctic region. In tundra zone of Russia: northern part of Western Siberia (Lukin 1976; Zaloznyj 1984).

########### Location.

There is no specimen in our collection from the Kharbey area.

########### Ecology.

This species is rare or absent in the northwestern part of Russia. Usually, it inhabits stagnant waters.

########## 67. Erpobdella

Taxon classificationAnimaliaArhynchobdellidaErpobdellidae

sp. 1

5A008472-F43C-5C33-A9F3-803321380CD6

########### Morphology.

All specimens had dark dorsal pigmentation with clearly defined two paramedian stripes and three annuli between sexual pores. This combination of morphological and anatomical features has not been found in any known species.

########### Location.

A small nameless lake near Syattey-ty (67°33'46.2"N, 62°41'32.8"E).

########### Ecology.

Multiple specimens were found in silt among *Arcticophila* thickets.

####### Suborder Hirudiniformes (Caballero, 1952)

######## Family Haemopidae (Richardson, 1969)

######### Genus *Haemopis* (Savigny, 1822)

########## 68. Haemopis sanguisuga

Taxon classificationAnimaliaArhynchobdellidaHaemopidae

(Linnaeus, 1758)

096FDBD9-80F3-5B36-9D51-C9C14E179A6A

Hirudo sanguisuga Linnaeus, 1758
Haemopis sanguisuga Blanchard, 1894

########### Geographic distribution.

Transpalearctic species. Widespread in all Europe and Asia up to Far East. In tundra zone on Russia: Lukin describes them as characteristic for Northern Eurasia (1976) and specifically for Komi Republic water bodies (1957).

########### Location.

There is no specimen in our collection from the Kharbey area.

########### Ecology.

This so called "large false horse leech" is a predator and lives mainly in shallow ponds, occasionally in temporary ponds where sediments remain wet; it is found only in the shore zone.

## Supplementary Material

XML Treatment for Amphichaeta leydigi

XML Treatment for Arcteonais lomondi

XML Treatment for Bratislavia palmeni

XML Treatment for Chaetogaster diaphanus

XML Treatment for Chaetogaster diastrophus

XML Treatment for Chaetogaster setosus

XML Treatment for Nais alpina

XML Treatment for Nais barbata

XML Treatment for Nais behningi

XML Treatment for Nais bretscheri

XML Treatment for Nais communis

XML Treatment for Nais elinguis

XML Treatment for Nais pardalis

XML Treatment for Nais pseudobtusa

XML Treatment for Nais simplex

XML Treatment for Nais variabilis

XML Treatment for Piguetiella blanci

XML Treatment for Ripistes parasita

XML Treatment for Slavina appendiculata

XML Treatment for Specaria josinae

XML Treatment for Stylaria fossularis

XML Treatment for Stylaria lacustris

XML Treatment for Uncinais uncinata

XML Treatment for Vejdovskyella comata

XML Treatment for Vejdovskyella macrochaeta

XML Treatment for Pristina aequiseta

XML Treatment for Pristina amphibiotica

XML Treatment for Pristina bilobata

XML Treatment for Pristina komi

XML Treatment for Pristina longiseta

XML Treatment for Rhyacodrilus coccineus

XML Treatment for Aulodrilus limnobius

XML Treatment for Aulodrilus pigueti

XML Treatment for Aulodrilus pluriseta

XML Treatment for Embolocephalus velutinus

XML Treatment for Ilyodrilus templetoni

XML Treatment for Isochaetides michaelseni

XML Treatment for Limnodrilus hoffmeisteri

XML Treatment for Limnodrilus udekemianus

XML Treatment for Potamothrix hammoniensis

XML Treatment for Spirosperma ferox

XML Treatment for Lophochaeta ignota

XML Treatment for Tubifex tubifex

XML Treatment for Alexandrovia ringulata

XML Treatment for Cognettia glandulosa

XML Treatment for Cognettia sphagnetorum

XML Treatment for Mesenchytraeus armatus

XML Treatment for Lumbriculus alexandrovi

XML Treatment for Lumbriculus variegatus

XML Treatment for Stylodrilus heringianus

XML Treatment for Eiseniella tetraedra

XML Treatment for Acanthobdella peledina

XML Treatment for Glossiphonia complanata

XML Treatment for Glossiphonia verrucata

XML Treatment for Glossiphonia concolor

XML Treatment for Hemiclepsis marginata

XML Treatment for Helobdella stagnalis

XML Treatment for Theromyzon tessulatum

XML Treatment for Theromyzon maculosum

XML Treatment for Piscicola geometra

XML Treatment for Piscicola

XML Treatment for Cystobranchus mammillatus

XML Treatment for Erpobdella octoculata

XML Treatment for Erpobdella monostriata

XML Treatment for Erpobdella nigricollis

XML Treatment for Erpobdella testacea

XML Treatment for Erpobdella

XML Treatment for Haemopis sanguisuga
